# Spent sulfuric acid plant catalyst: valuable resource of vanadium or risky residue? Process comparison for environmental implications

**DOI:** 10.1007/s11356-020-11349-z

**Published:** 2020-10-27

**Authors:** Bartosz Mikoda, Anna Potysz, Agnieszka Gruszecka-Kosowska, Ewa Kmiecik, Anna Tomczyk

**Affiliations:** 1 Faculty of Geology, Geophysics and Environmental Protection, AGH University of Science and Technology, al. A. Mickiewicza 30, 30-059 Krakow, Poland; 2 Institute of Geological Sciences, University of Wrocław, Cybulskiego 30, 50-205 Wrocław, Poland

**Keywords:** Vanadium, Metal recovery, Waste management, Environmental risk, Secondary resources, Sustainable development, Biohydrometallurgy

## Abstract

The enormous amount of spent catalysts generated worldwide may pose a risk to the environment because of their high load of metals, including vanadium. The latter may be mobilized and released to the environment if managed improperly. Moreover, the catalysts could be considered as secondary resources rather than waste. This study aimed at the efficient extraction of vanadium from spent desulfurization catalyst (SDC) from a sulfuric acid production plant. The raw SDC and the post-extraction residues were characterized in terms of their chemical and phase composition. The metal mobility from the materials was examined with both single-step and multi-step extractions. The environmental risk assessment was performed using sequential extraction. The study revealed that both tested methods (citric acid leaching and bioleaching with *Acidithiobacillus thiooxidans*) enable the extraction of nearly 96% of V from SDC with a simultaneous reduction of metal mobility. However, the bacterial treatment was found more suitable. The leached residue was mostly (> 90%) composed of SiO_2_, which makes it a potential candidate for application in construction (e.g., concrete mixtures) after additional examinations. The study highlights the need to develop a metal extraction process for SDC in a way that metal-free residue could be a final product.

## Introduction

Having a high-quality living environment is of great importance for humans. However, industrial development deteriorates the environment, which can lead to harmful effects on living organisms, including humans (Mikoda et al. 2017). One method that ensures both high-quality products and a cleaner environment is catalysis (from Greek *katalein*—decompose). In this process, catalysts (substances that modify chemical reactions without undergoing any reactions themselves—mostly solid-state) are used to remove undesired constituents from the mixture of interest (Alvarez-Amparán and Cedeño-Caero 2017). Various reactions are prompted with the use of catalysts, such as cleaning engine fumes, processing heavy crude oil, and oxidation or reduction of given elements (e.g., sulfur, nitrogen) in flue gas emissions. Different applications require different chemical compositions and unique properties. However, some common features such as high specific surface area and the presence of an active compound (usually metals, for example Co, Mo, Ni, V, W, Pt, Pd) doped on a support phase (e.g., SiO_2_, Al_2_O_3_, TiO_2_) (Marafi et al. 2017; Cecilia et al. 2018). Catalysts have different life cycles (1–2 years for desulfurization, 3–5 years for nitrogen reduction), which can be shortened by factors such as the formation of coke, precipitation of metal salts, or adsorption of organic compounds. These factors either reduce the number of active sites or plug the pore system (Vogelaar et al. 2010; Maity et al. 2012). Eventually, the catalysts become unusable and must be replaced. The spent catalysts are often loaded with metals, oil, or coke, which makes them hazardous and hampers their use. Many are disposed of on sites dedicated to hazardous waste (Marafi and Stanislaus 2008). The spent catalysts generated worldwide include 700,000 to 900,000 tons of spent fluid catalytic cracking (FCC) residues (Muddanna and Baral 2019); up to 200,000 tons of catalyst from petroleum industry (Pathak et al. 2018); up to 40,000 tons of catalyst from sulfuric acid production (Nikiforova et al. 2017); and up to 38,000 tons of selective catalytic reduction (SCR) waste in China itself (Dai et al. 2018). These numbers force researchers to find comprehensive methods of economic and environment-friendly use of catalysts.

Waste materials from catalytic activities are important as prospective secondary source of metals (Pathak et al. 2014; Akcil et al. 2015). These residues may be used after metal recovery if environmental standards are met. For many years, research efforts have been undertaken to find efficient solutions for catalyst utilization. Two methods that have been considered for this were deoiling or decoking and metal extraction or recovery (Al-Sheeha et al. 2013; Wiecka et al. 2020). Using unprocessed spent catalysts in the production of new catalysts or using them as additives to other useful materials also has been explored through the years (Furimsky and Biagini 1996; Trochez et al. 2015; Marafi et al. 2017). However, it is not always possible to remove oil, coke, or metals from the catalyst. In that case, the material becomes waste and it must be discarded.

Most methods for removing metals from spent catalysts are physical (pyrometallurgy), chemical (hydrometallurgy), or biological (bioleaching). Pyrometallurgical methods are mainly used to process automotive catalysts to recover noble metals such as Pt or Pd (Peng et al. 2017). However, pyrometallurgy has huge disadvantages, namely the need for special equipment and high energy consumption. Moreover, concentrated metals must be refined (Ding et al. 2019; Pathak et al. 2020; Wiecka et al. 2020). Therefore, the application of high-temperature treatment is limited from both environmental and economic viewpoints. Recently, (bio)leaching with chemical and bacterial means using a plethora of lixiviants and bacterial strains has attracted a great deal of attention from researchers worldwide (Asghari et al. 2013; Pradhan et al. 2013; Srichandan et al. 2014; Vyas and Ting 2016; Vyas and Ting 2018; Pathak et al. 2020; Le and Lee 2020). Both chemical and biological leaching procedures led to high yields of released metals (even up to 100%) in both single-step and multi-step processes, and in combined chemical-biological processes. The metals in pregnant leach solutions may be either chemically precipitated or recovered using ion-exchange resins (Mazurek 2013; Innocenzi et al. 2014; Sethurajan et al. 2016; Pradhan et al. 2020).

The “raw” spent catalysts pose a threat to the environment because metals concentrated in these wastes are in high amounts and in mobile forms (Dai et al. 2018). Treating these wastes can significantly reduce the mobility of metals, which Pathak et al. (2014, 2018) proved using chemical leaching and bioleaching. Biological treatment reduced the mobility of metals, so that most of them (98–99%) were in residual fraction after four-step sequential extraction. (Bio)hydrometallurgical treatment not only causes the recovery of metals present in catalysts but also enhances the environmental stability of these wastes, which is more encouraging in terms of their further application.

The novelty of this study is mainly related to “green” approach applied that was a treatment generating residue depleted in vanadium. The element was recovered as a value. It has to be emphasized that recently the European Union places a great focus to supply raw materials. The EU economy and the list of critical raw materials have been recently upgraded (European Commission 2020). Among the raw materials identified by the European Commission, vanadium is included. Therefore, the current action policy adopted by the EU is oriented to foster raw material recycling and to promote research on recovery of raw materials. In these regards, sustainable waste management requires an extension of life cycle of product, meaning that it encourages to collect the spent desulfurization catalyst and further treat it as secondary resource rather than considering it as end-of-life product.

This study was aimed to define an efficient method to extract vanadium from spent desulfurization catalyst (SDC) from the sulfuric acid plant and to characterize post-(bio)leaching residues in terms of environmental risk. Leaching of V was carried out using organic lixiviant (citric acid (CA)) and bacterial strain *Acidithiobacillus thiooxidans* (AT). The standardized protocols (both single-step and multi-step) were applied to assess the environmental stability of both raw SDC and leached residues.

## Materials and methods

### Characterization of raw and (bio)leached catalyst

**Fig. 1:**
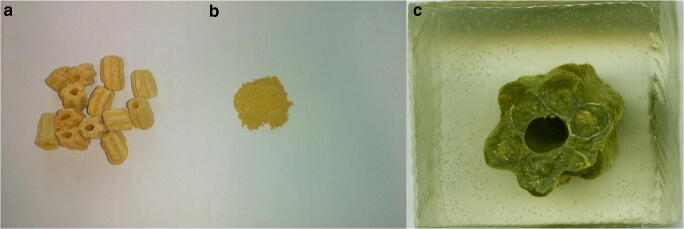
Photographs of SDC samples: as-received (**a**), comminuted and sieved (**b**), and polished section (**c**)

The SDC sample was received from a sulfuric acid production plant, which is a unit of a copper smelter. The catalyst is used there to oxidize SO_2_ to SO_3_, which is later utilized for H_2_SO_4_ production. The as-received sample consisted of 10-mm yellow granules (Fig. 1a). A polished section was created on one granule (Fig. 1c), which was later subjected to microscopic observations using an optical microscope with reflected light (Nikon Eclipse LV100POL). Subsequently, selected areas were examined using a scanning electron microscope (FEI Quanta 200 FEG) coupled with an EDAX energy-dispersion spectrometer (SEM-EDS). The operating parameters were as follows: low vacuum mode, 20 kV accelerating voltage, 10 nA beam current, 50 s counting time, 34° take-off angle.

The phase and chemical characteristics of raw and leached residues were determined using X-ray diffraction (XRD) and X-ray fluorescence (XRF), respectively. XRD pattern was created with the Rigaku MiniFlex 600 device, on 0.5 g sample comminuted to < 5 μm, with the following working parameters: Cu-Kα radiation, 3–75° 2*θ* range, 0.05° step width. The XRD results were processed with XRAYAN software coupled with PDF-4 database. The chemical oxide composition was determined using the Rigaku ZSX Primus II apparatus (X-ray tube: end-window, with Rh anode; 4 kW power) from 4.5 g sample powdered to < 100 μm mixed with 1.5 g of CELLEOX® binding aid to form a pellet. The wave intensities were calculated to oxide composition using the fundamental parameter method.

Specific surface area (SSA) of raw and leached residues was determined using the N_2_ adsorption/desorption method in 77 K temperature with Micromeritics ASAP 2020 apparatus. 0.5 g portions of each residue were heated at 105 °C for 12 h before taking the measurements. The Brunauer-Emmett-Teller (BET) isotherm was used to characterize the specific surface area of the examined materials.

### Vanadium extraction

The SDC sample has been comminuted (Fig. 1b) and sieved using two sets of sieves (0.1 mm and 0.2 mm) to investigate the effect of particle size on leaching efficiency. The citric acid (CA) solutions (0.1 M and 1 M) were used to evaluate the impact of acid concentration on V leaching. The third parameter used to optimize the process was pulp density (PD), which was evaluated in the range between 1 and 20%. The extraction rate was analyzed in the time frame of 8–72 h. The orbital shaker was set at 150 rpm speed.

The bacterial extraction was performed using the gram-negative bacterial strain *Acidithiobacillus thiooxidans* (AT; DSM 9463) provided by Leibniz-Institut DSMZ (Deutsche Sammlung von Mikroorganismen und Zellkulturen GmbH). The AT strain was cultivated in a medium consisting of (all reagents of analytical grade, Merck, Germany) 2 g (NH_4_)_2_SO_4_, 0.25 g MgSO_4_ ∙ 7H_2_O, 0.1 g K_2_HPO_4_, 0.1 g KCl, and 1% (w/v) elemental sulfur per 1 dm^3^ of ultrapure water (Millipore, Milli-Q), with pH adjusted to 2.5 using 10 M H_2_SO_4_ (Potysz et al. 2020). The biotic experiments were conducted in Erlenmeyer flasks, where portions of SDC were mixed with 50 cm^3^ of fresh medium, 2% (w/v) of elemental S, and 2% (v/v) of bacterial inoculum. The abiotic tests were also conducted identically, except from the inoculum addition. Experiments were extended from 1 to 28 days using pulp densities (PD) of 1 to 20%.

An earlier study (Mikoda et al. 2020) revealed that the optimal extraction conditions were 100–200 μm particle size (for both extractions), 48 h time and 10% PD (CA), and 21 days’ time and 2% PD (AT), giving V extraction yields of 95% (CA) and 93% (AT). Therefore, these conditions were applied again to get the materials for this study.

### Environmental risk assessment study

#### Leaching of elements with simulated rainfall

The raw and leached SDC samples were subjected to leaching tests using standardized environmental protocols accepted worldwide. The discharge of metals from SDC samples when exposed to artificial rainfall was assessed using the EPA 1312 SPLP test. In this experiment, 1 g of each residue was put into separate 50 cm^3^ Falcon tubes filled with 20 cm^3^ of ultrapure water, acidified to pH 4.20 ± 0.05 using a mixture of 95% H_2_SO_4_ and 65% HNO_3_ (60:40 volume ratio). The samples were shaken for 18 h (150 rpm speed), centrifuged, filtered with Whatman 0.45 μm syringe filters, acidified with 0.1 cm^3^ of 65% HNO_3_, and stored at 4 °C for analysis.

#### Forms of metal occurrence in raw and (bio)leached catalyst

The fractionation of metals in raw and leached SDC materials was evaluated using a four-step sequential extraction protocol, stemming from a three-step procedure proposed by BCR (Pueyo et al. 2008). BCR extraction causes the release of four fractions (Table 1): exchangeable F1 (leached with 0.11 M acetic acid) which represents adsorbed metals easily affected by the ionic composition of water; reducible F2 (leached with 0.11 M hydroxylamine hydrochloride with pH adjusted to 2) which corresponds to metals affected by anoxic conditions; oxidizable F3 (leached with 30% hydrogen peroxide and 1 M ammonium acetate with pH adjusted to 2), corresponding to metals released during highly oxic conditions, bound to organic matter and/or sulfides; and residual F4 (digested with aqua regia), representing metals bound to the crystal lattice of the material. The analysis of metal binding forms was conducted on 1 g sample of each material, mixed with the lixiviant for a given time (Table 1) in 50 cm^3^ Falcon polypropylene tubes. The shaking speed was 150 rpm. The tubes were centrifuged after the extraction, and the liquid samples were filtered with Whatman 0.45 μm syringe filters, acidified with 0.1 cm^3^ of 65% HNO_3_, and stored in 4 °C for analysis. The solid samples were washed with ultrapure water and subjected to the subsequent extraction step. The first part of the 3rd step (H_2_O_2_ leaching) was conducted on a water bath. The 4th step (aqua regia digestion) was performed using SCP Science DigiPrep HT 250 apparatus (Quebec, Canada) with 250 cm^3^ glass tubes. The liquid samples from the 4th step were diluted to 50 cm^3^ with ultrapure water, filtered using paper filters, and then subjected to the same procedure as the other liquid samples.

**Table 1:** Parameters of sequential extraction with targeted fractions according to modified BCR procedure (Pueyo et al. 2008)

Step	Chemical agent	pH	SSR	Conditions	Time (h)	Fraction
I	40 cm^3^ 1 M C_2_H_4_O_2_	3	1:40	Shaking (RT)	16	Exchangeable (F1)
II	40 cm^3^ 0.1 M [NH_3_OH]Cl	2	1:40	Shaking (RT)	16	Reducible (F2)
IIIa	2x10 cm^3^ 30% H_2_O_2_	ns	1:10	Heating (85 °C)	2 × 1	Oxidizable (F3)
IIIb	50 cm^3^ 1 M C_2_H_7_NO_2_	2	1:50	Shaking (to complete the reaction)	16	Oxidizable (F3)
IV	10 cm^3^ aqua regia	ns	1:10	Digestion (130 °C)	2	Residual (F4)

*RT*, room temperature; *SSR*, sample-to-solvent ratio; *ns*, not specified

#### Environmental risk indices

The results of the sequential extraction procedure were used to evaluate the potential environmental risk of raw and leached SDC residues. In this study, a reduced partition index (*I*_R_) and a risk assessment code (RAC) were used to assay the potential environmental risk posed by the spent catalyst before and after (bio)leaching.

The *I*_R_ is a commonly used index since it gives an insight into the relative binding intensity of an element in a solid matrix (Miretzky et al. 2011). *I*_R_ is defined by (Eq. 1):

1 $$ {I}_{\mathrm{R}}=\sum \limits_{i=1}^k{i}^2 Fi/{k}^2\kern0.5em $$

where *i* is the index number of the BCR sequential extraction step, *k* = 4 (total steps in BCR sequential extraction), and *Fi* is the percentage of a particular metal in fraction *i*. The values of *I*_R_ are between 0.06 and 1.00. The lower the *I*_R_ value, the weaker the binding of a metal, which corresponds to higher mobility.

The RAC index is indirectly related to *I*_R_ (the higher the RAC, the lower the *I*_R_) and reflects the mobility of metals (Gusiatin and Kulikowska 2014). It is defined by (Eq. 2):

2 $$ \mathrm{RAC}=\frac{C_{\mathrm{m}}}{C_{\mathrm{total}}}\times 100\% $$

where *C*_m_ is a metal concentration in exchangeable fraction and *C*_total_ is the total concentration of the given metal. Based on the F1 fraction, the RAC can have values from 1 to 100%: < 1% (no risk), 1 to 10% (low risk), 11 to 30% (medium risk), 31 to 50% (high risk), and > 50% (very high risk) for a given metal.

### Analysis of vanadium content in liquid samples

The solutions, filtered and acidified (according to the procedure described in each section), were analyzed using inductively coupled plasma mass spectrometry (ICP-MS; Perkin Elmer ELAN 6100 device) according to the ISO 17294-2:^2016^ protocol. The apparatus was calibrated using multielement ICP standards provided by Merck (Germany). The QA/QC was performed by analyzing blank and duplicate samples and duplicate analysis of randomly chosen samples. The limits of quantification for the specific elements were as follows (in mg dm^−3^): Al: 0.005, Fe: 0.02, K: 0.05, Mg: 0.001, Na: 0.01, S: 0.1, and V: 0.001. The results were multiplied by a liquid-to-solid ratio to express the leaching yields in mg per kg of catalyst.

### Data processing

The results from the liquid samples were processed using MS Excel 2016 software. The environmental risk indices also were obtained from calculations in MS Excel 2016. The graphs were prepared from the Excel files using Grapher 11 software.

## Results and discussion

### Characterization of raw spent catalyst and (bio)leaching residues

**Fig. 2:**
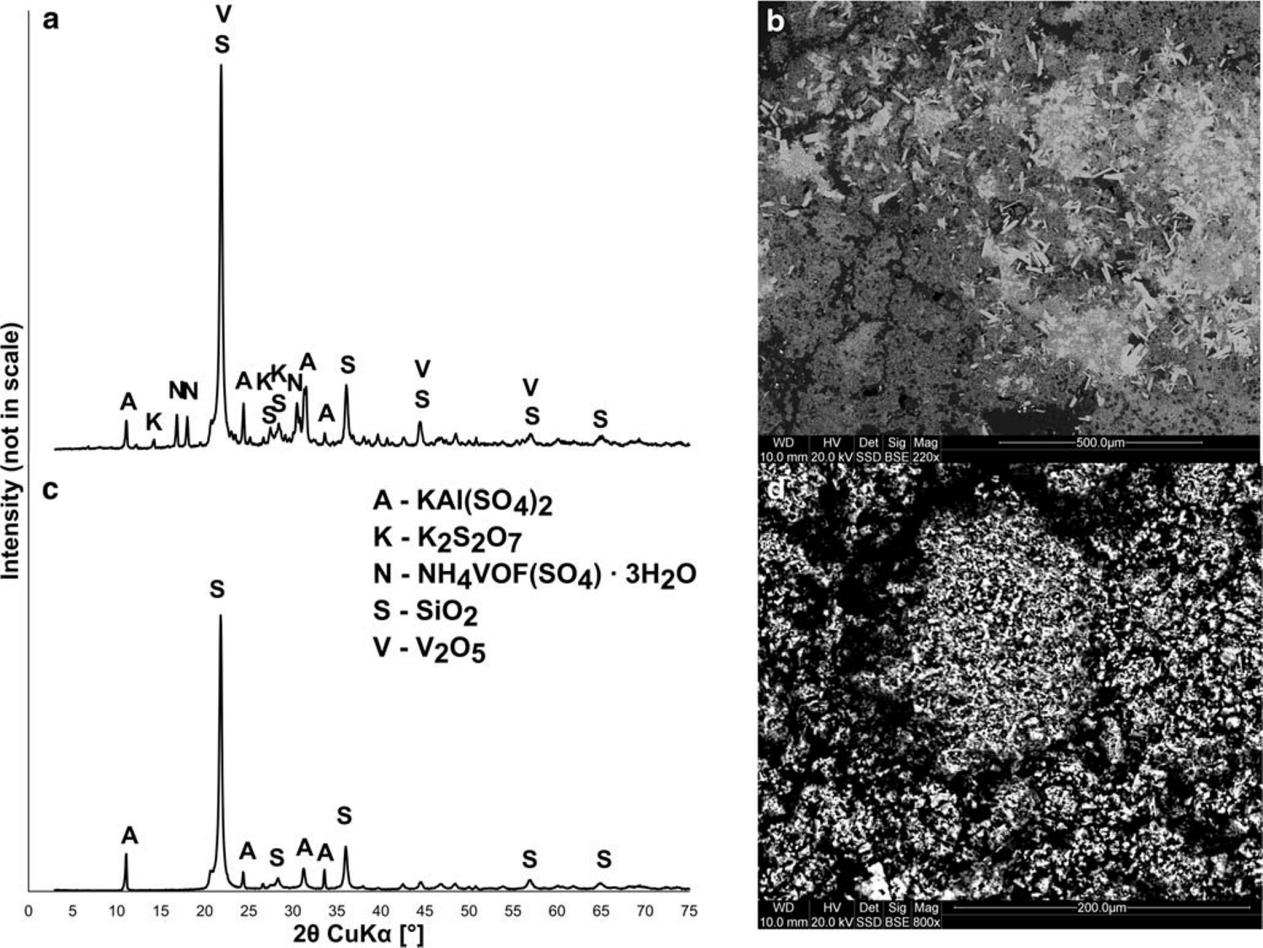
**a** XRD pattern of raw SDC sample. **b** SEM photomicrograph of raw SDC sample, showing porous SiO_2_ matrix (dark gray) and KAl(SO_4_)_2_ euhedral crystals (light gray). **c** XRD pattern of leached SDC sample. **d** SEM photomicrograph of leached SDC sample, showing mixed SiO_2_ matrix with metal remnants

The results of phase analysis by XRD (Fig. 2a) showed that the main phase in the raw SDC sample was SiO_2_ in the form of cristobalite or tridymite, which is typically used as a porous support phase. Additional phases comprised KAl(SO_4_)_2_, K_2_S_2_O_7_, NH_4_VOF(SO_4_) ∙ 3H_2_O, and V_2_O_5_. The last two phases corresponded to potassium pyro-sulfo-vanadate, doped on the porous surface of the catalyst. SEM observations confirmed the existence of a porous support phase built of SiO_2_ and doped with V salts. Some euhedral crystals of KAl(SO_4_)_2_ also were embedded in the porous matrix (Fig. 2b). The composition was typical for catalysts used for oxidative desulfurization of flue gases (Mazurek 2013; Nikiforova et al. 2016). The XRD results of post-leaching residues showed that the sole remaining phases after CA and AT leaching were SiO_2_ polymorphs and KAl(SO_4_)_2_, while V-containing phases disappeared (Fig. 2c). SEM observations of post-leaching residues showed that the remaining V and other elements were mixed with the support of a SiO_2_ phase (Fig. 2d).

The chemical composition of raw SDC material (Table 2) showed that the dominant component of the raw SDC was SiO_2_. The other important constituents were SO_3_, K_2_O, V_2_O_5_, Al_2_O_3_, Na_2_O, Fe_2_O_3_, and MgO. The remaining 0.4% of raw SDC consisted of elements such as Ca, P, Ti, and Cl and other minor contaminants (data not shown). The chemical composition of CA-and AT-leached SDC samples (Table 2) revealed not only that V was leached to the solution but also that the concentrations of other elements were reduced compared to raw SDC. A large decrease was noted for V (ca. 96% for both materials; Table 2). This was essential from the viewpoint of further process implementation and up-scaling. The big reduction of concentration was also noted for Na, Mg, S, and K, whereas the reduction of Fe and Al was smaller. The Si content increased from 65.5% SiO_2_ to 92% (CA) and 96.5% (AT). The more efficient leaching of elements was noted for AT treatment, except for V (Table 2).

**Table 2:** Chemical composition (determined by WDXRF) and specific surface area (SSA; determined by BET method) of raw, acid-leached, and bioleached SDC samples

Chemical compounds (wt.%)	Raw SDC	Acid-leached SDC	Bioleached SDC
Na_2_O	1.64	0.08	0.05
Al_2_O_3_	1.67	1.53	0.51
SiO_2_	65.5	92.0	96.5
SO_3_	15.5	4.10	2.20
K_2_O	8.77	1.33	0.35
V_2_O_5_	5.50	0.19	0.22
Fe_2_O_3_*	0.73	0.52	0.13
MgO	0.23	0.06	0.02
Total	99.6	99.8	100
SSA [m^2^ g^−1^]	2.34	2.86	3.51

*Total Fe

The BET-calculated SSA of raw and leached SDC (Table 2) showed that (bio)leaching increased the SSA of the examined residue. The SSA values were 2.34 m^2^ g^−1^ for raw SDC, 2.86 m^2^ g^−1^ for CA-leached SDC, and 3.51 m^2^ g^−1^ for AT-leached SDC.

### Metal fractionation in spent catalyst before and after (bio)leaching

**Fig. 3:**
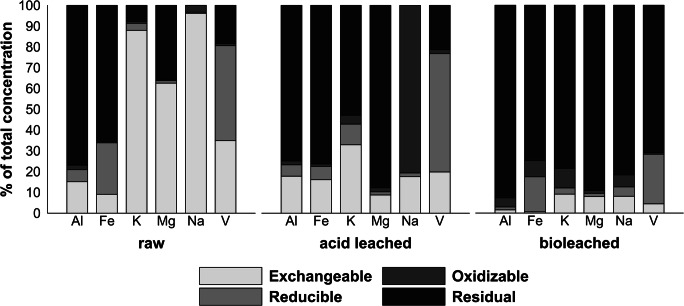
Metal fractionation in raw, acid-leached, and bioleached SDC samples

The results of the sequential extraction procedure (Fig. 3) revealed significant differentiation in metal fractionation before and after SDC treatment. For the raw SDC sample, elements such as K, Mg, Na, and V were present mostly in exchangeable forms (88%, 64%, 96%, and 81%, respectively), whereas Al and Fe were present mostly in a residual form (77% and 66%, respectively). In the CA-leached sample, much lower amounts of metals were in a mobile form (33% K, 9% Mg, 18% Na, and 20% V, respectively). The exceptions were Al and Fe, which exhibited higher mobility (18% Al vs 15% prior to leaching, 16% Fe vs 9% prior to leaching). The AT treatment of SDC reduced the percentage of metals in mobile forms to an even greater extent (all elements < 10% in exchangeable fraction and < 28% in combined exchangeable/reducible fractions). Of these, the most abundant metals in F1–F2 fractions were Fe and V (18% and 27%, respectively). In addition, the AT treatment of SDC transformed the examined elements into residual fractions (93% Al, 75% Fe, 79% K, 89% Mg, 82% Na, and 71% V). The only element present in F1–F3 fractions in raw and CA-leached samples was Na. During AT treatment, it was transferred mostly into residual fraction, which may be caused by forming clathrates and returning metals into the solid phase (Dai et al. 2018). The results showed a good agreement with earlier studies of Pathak et al. (2014, 2018) which showed that bioleaching spent refinery catalysts with a chemolithoautotrophic bacterium (*Acidithiobacillus ferrooxidans*) also could significantly reduce mobile forms of metals in this type of waste.

### Environmental risk assessment

The SPLP leaching test results (Table 3) suggested that, compared to Polish wastewater regulations (RMMAIN 2019), the concentrations of the analyzed elements were exceeded, except for Na. This method evaluates the worst-case scenario for waste landfilling (Mikoda and Gruszecka-Kosowska 2018; Mikoda et al. 2019), so in this view, both raw and leached residues posed a threat to the water environment due to exceeded contents of substances particularly harmful to the aquatic environment in leachates. The load of harmful substances was much smaller in the case of CA-leached SDC (except for Al and Fe). In contrast, the AT treatment significantly reduced the leachable forms of metals compared to artificial rainfall-sample contact (Table 3), leaving only Al and V in amounts that exceed regulatory values. This was another proof that bioleaching was more efficient at reducing the environmental impact of the spent catalyst.

**Table 3:** Results of SPLP test for selected elements of raw, acid-leached, and bioleached SDC samples and Polish regulatory values of these elements (RMMAIN 2019)

SDC sample	Concentration [mg dm^−3^]					
	Al	Fe	K	Na	S	V
Raw	63	28	2941	589	4746	438
Acid leached	72	28	175	41	575	10
Bioleached	4.8	0.5	0.8	2.2	113	6.4
Regulatory value	3	10	80	800	200*	2

*Calculated from value expressed as SO_3_ (500 mg dm^−3^)

Reduced partition indices (*I*_R_; Table 4) confirmed the decrease of risk for metals such as K and Mg, expressed with higher binding strength (*I*_R_ closer to 1), when treated with CA. For Na and V, the *I*_R_ values increased, but remained relatively low. The values for Al and Fe remained virtually constant, but close to 1. This showed their high binding affinity in both materials. In the case of AT leaching, *I*_R_ values for all metals were greater than 0.77 (Table 4). This showed that their mobility was visibly decreased by bacterial leaching.

**Table 4:** Reduced partition indices (*I*_R_) and risk assessment code (RAC) values of selected metals for raw, acid-leached, and bioleached SDC samples

Indicator	SDC sample	Unit	Al	Fe	K	Mg	Na	V
*I*_R_	Raw	-	0.80	0.73	0.15	0.40	0.07	0.33
	Acid leached		0.78	0.80	0.60	0.90	0.33	0.38
	Bioleached		0.96	0.83	0.85	0.91	0.87	0.78
RAC	Raw	%	15	9	88	63	96	35
	Acid leached		18	16	33	9	18	20
	Bioleached		2	1	9	8	8	4

RAC values for both materials suggested that the risk was significantly lower for CA-leached SDC compared to raw SDC (Table 4). This was most visible for K (risk decreased from very high to high), Mg (risk decreased from very high to low), and Na (risk decreased from very high to medium). In the case of AT treatment, risk values dropped notably to low risk (for all elements) or even to the boundary of low risk/no risk for Fe (Table 4).

The present study revealed that risk indices were very good tools for expressing the environmental threat posed by waste materials and for illustrating the changes in metal fractionation profiles during treatment. The usability of risk indices in waste management and environmental science as a whole also was confirmed in other studies (Miretzky et al. 2011; Innocenzi et al. 2014; Pathak et al. 2018).

### Perspectives on the potential use of raw and (bio)leached catalyst

The spent desulfurization catalyst from the copper smelting facility has the potential for further utilization, especially after bioleaching, since risk indices were low and mobile fractions of the remaining elements were scarce. The composition of all waste materials was dominated by SiO_2_, which suggests these materials could be used in construction engineering, e.g., as activators in cement materials (Simonsen et al. 2020). However, the amount of Al_2_O_3_ is too small to use these wastes as geopolymers (Trochez et al. 2015). The high amount of V present in the untreated material can be extracted efficiently with citric acid and *Acidithiobacillus thiooxidans* bacteria (up to 96%; see Table 2). Both materials (raw and CA leached), however, pose a threat to the environment, because metals such as K, Mg, Na, and V are easily mobilized, as demonstrated by sequential extraction, especially from raw SDC (Table 3; Table 4). On the other hand, the AT-leached residue was much safer in terms of the extracted amounts of elements (Table 3; Fig. 3) and the posed risk (Table 4). In addition, the matter of using metal-free residue must be examined in the future. When comparing the specific surface area values of other spent catalysts (17–205 m^2^ g^−1^; Furimsky and Biagini 1996) with the ones from this study (Table 2), one can see that the SSA of raw and leached residues is very small, which is rather undesirable in the case of using the residues as a basis for new catalysts. Theoretically, high SiO_2_ content (> 90%; Table 2) predestines the leached residues to be considered as sand replacement in concretes. Al-Jabri et al. (2013) showed that spent catalysts can be used as partial sand replacement in cement mortars, but those catalysts had high Al_2_O_3_ content, which was lacking in the SDC residues from this study. Other studies have shown that high SiO_2_ materials such as glass powder or waste foundry sand can be successfully used to replace sand in concrete mixtures (Islam et al. 2017; Mavroulidou and Lawrence 2019). That application, however, requires additional research on the physical parameters of the residue, such as specific gravity and moisture. Nonetheless, detailed studies of the influence of raw and leached SDC addition to concrete mixtures should be conducted to provide answers for unsolved questions about SDC utilization.

## Conclusions

The present study showed that both single-step citric acid leaching and *Acidithiobacillus thiooxidans* bioleaching were proven efficient for metal extraction from spent desulfurization catalyst from a sulfuric acid plant. However, based on risk indices, the CA-leached catalyst might pose environmental risks, whereas the AT-leached residue created much less concern. Because biological treatment reduces metal mobility in spent catalysts, we recommend using bioleaching as an additional step in the extraction procedure or using bioleaching itself as the main method. Further utilization was hampered by low specific surface area of all examined residues, which excludes those to be used as new catalysts. However, high SiO_2_ content predestines the materials for further assessment, e.g., as an additive to cement mixtures.

## Data Availability

Not applicable.
